# Long-Term Results of Mitral Valve Repair

**DOI:** 10.21470/1678-9741-2017-0145

**Published:** 2018

**Authors:** Francisco Diniz Affonso da Costa, Daniele de Fátima Fornazari Colatusso, Gustavo Luis do Santos Martin, Kallyne Carolina Silva Parra, Mariana Cozer Botta, Eduardo Mendel Balbi Filho, Myrian Veloso, Gabriela Miotto, Andreia Dumsch de Aragon Ferreira, Claudinei Colatusso

**Affiliations:** 1 Pontifícia Universidade Católica do Paraná (PUC-PR), Curitiba, PR, Brazil.; 2 Department of Cardiac Surgery, Instituto de Neurologia e Cardiologia de Curitiba (INC-Cardio), Curitiba, PR, Brazil.

**Keywords:** Mitral Valve, Mitral Valve Insufficiency, Mitral Valve Prolapse, Mitral Valve Annuloplasty

## Abstract

**Introduction:**

Current guidelines state that patients with severe mitral regurgitation should be treated in reference centers with a high reparability rate, low mortality rate, and durable results.

**Objective:**

To analyze our global experience with the treatment of organic mitral regurgitation from various etiologies operated in a single center.

**Methods:**

We evaluated all surgically treated patients with organic mitral regurgitation from 2004-2017. Patients were evaluated clinically and by echocardiography every year. We determined early and late survival rates, valve related events and freedom from recurrent mitral regurgitation and tricuspid regurgitation. Valve failure was defined as any mitral regurgitation ≥ moderate degree or the need for reoperation for any reason.

**Results:**

Out of 133 patients with organic mitral regurgitation, 125 (93.9%) were submitted to valve repair. Mean age was 57±15 years and 52 patients were males. The most common etiologies were degenerative disease (73 patients) and rheumatic disease (34 patients). Early mortality was 2.4% and late survival was 84.3% at 10 years, which are similar to the age- and gender-matched general population. Only two patients developed severe mitral regurgitation, and both were reoperated (95.6% at 10 years). Freedom from mitral valve failure was 84.5% at 10 years, with no difference between degenerative and rheumatic valves. Overall, late ≥ moderate tricuspid regurgitation was present in 34% of the patients, being more common in the rheumatic ones. The use of tricuspid annuloplasty abolished this complication.

**Conclusion:**

We have demonstrated that mitral regurgitation due to organic mitral valve disease from various etiologies can be surgically treated with a high repair rate, low early mortality and long-term survival that are comparable to the matched general population. Concomitant treatment of atrial fibrillation and tricuspid valve may be important adjuncts to optimize long-term results.

**Table:** 

Abbreviations, acronyms & symbols				
ACC	= American College of Cardiology		MD	= Myxomatous degeneration
AF	= Atrial fibrillation		MR	= Mitral regurgitation
AVR	= Aortic valve replacement		MVR	= Mitral valve repair
CABG	= Coronary artery bypass grafting		NYHA	= New York Heart Association
CAD	= Coronary artery disease		RA	= Right atrial
CI	= Confidence intervals		RD	= Rheumatic disease
COPD	= Chronic obstructive pulmonary disease		RV	= Right ventricular
CPB	= Cardiopulmonary bypass		SAM	= Systolic anterior motion
DDLV	= Diastolic dimension of the left ventricle		SD	= Standard deviation
ECC	= Extended extracorporeal circulation		SDLV	= Systolic dimension of the left ventricle
FD	= Fibroelastic deficiency		SIH/SUS	= Hospital Information System - Ministry of Health
HR	= Hazard ratios		STS	= Society of Thoracic Surgeons
INC-Cardio	= Instituto de Neurologia e Cardiologia de Curitiba		TEE	= Transesophageal echocardiography
LV	= Left ventricle		TR	= Tricuspid regurgitation
LVEF	= Left ventricular ejection fraction		USA	= United States of America

## INTRODUCTION

Mitral valve repair (MVR) is considered the best surgical option for most patients with severe mitral regurgitation (MR), including elderly patients with associated comorbidities and higher operative risks^[1,2]^. In fact, recent guidelines for management of valvular heart diseases indicate that asymptomatic patients with preserved left ventricular function should be redirected to "reference centers" and surgery should be considered only when the probability of the repair is high, and the operative mortality is low^[3,4]^.

The Society of Thoracic Surgeons (STS) database demonstrates that despite an increasing adoption of conservative operations in the last decade, the overall rate of MVR in the United States of America (USA) is still only around 70%, and probably restricted to simpler repairs involving prolapse of the posterior cusp, in most centers^[5]^. In Brazil, MVR is performed even more infrequently, and although the statistical estimates are somewhat imprecise, the incidence of MVR appears to be in a disappointingly range of 1520% (Hospital Information System - Ministry of Health [SIH/SUS]).

Our surgical group, working in two different institutions, has been involved in mitral valve repair since the late 1980's. The Instituto de Neurologia e Cardiologia de Curitiba (INC-Cardio) is a new private health institution in Curitiba, and since its inception in 2004, we have had the opportunity to establish a heart valve team that enabled us not only to perform all valvular operations with intraoperative transesophageal echocardiography (TEE) control, but also to prospectively follow our patients at ambulatory level in an adequate manner. The aim of this study was to analyze our global experience with all surgically treated patients presented with any form of organic mitral valve insufficiency at this institution.

## METHODS

This study was approved by the Institutional Research Ethical Committee (number 1.061.245) and registered at Plataforma Brasil (number CAAE 42.708015.2.0000.5227). We retrospectively analyzed all patients operated at INC-Cardio from January 2004 to March 2017 who had the diagnosis of organic MR as the primary indication for surgery. Patients with concomitant procedures such as tricuspid repair, ablation for atrial fibrillation (AF), and coronary artery bypass grafting (CABG) were included. However, patients with concomitant aortic valve replacement (AVR) or functional MR due to ischemic cardiomyopathy were excluded from the analysis.

The etiology of the disease was based in echocardiographic findings and confirmed at the operation by visual inspection. Patients with degenerative disease were subdivided in fibroelastic deficiency (FD), moderate myxomatous degeneration (MD), and Barlow disease, according to the degree of myxomatous changes. Typically, patients with normally sized and thin cusps, except for the prolapsing segment, were considered FD; while those with voluminous, aneurysmal and thickened cusps, with massive annular dilatation and often displaced posterior cusp attachment, were categorized as Barlow disease. Valves with intermediary changes in this spectrum were labeled as MD. Rheumatic disease (RD) patients were included if they had pure MR or mixed lesions with at least moderate degree of MR.

### Surgical Technique

All operations were performed by a single surgeon (FDAC), most commonly through full sternotomy or, in the last few years, using a right minithoracotomy approach in selected cases. When midline sternotomy was used, cardiopulmonary bypass (CPB) was instituted with central aortic and bicaval cannulation. We routinely employed moderate systemic hypothermia (32ºC), and myocardial protection was obtained with intermittent antegrade cold blood cardioplegia. For the minithoracotomy cases, CPB was instituted with peripheral arterial and venous femoral cannulation, and cardioplegia was performed with a single dose of Custodiol solution^®^.

After gaining exposure of the mitral valve through a left atriotomy parallel to the interatrial groove, careful and systematic valve analysis was performed to identify all valve lesions producing valve dysfunction. Posterior leaflet prolapses were treated either by triangular or quadrangular resection of the prolapsing segments or by the insertion of neo-chords utilizing the "respect" concept. Whenever there was excess of posterior leaflet tissue, a sliding plasty was performed to reduce the height of the posterior leaflet in order to avoid systolic anterior motion (SAM). Anterior leaflet prolapse was preferentially corrected with the utilization of Gore-Tex neo-chords, but we also used the flipover technique or papillary muscle repositioning in some cases. In rheumatic cases, shaving and thinning of the leaflets as well as resection of scarred and fused primary and secondary chords were often necessary to increase leaflet mobility. Cusp extension of the anterior leaflet was utilized to increase surface coaptation in some cases. Mild or moderate areas of calcifications were fully debrided. In the presence of active bacterial endocarditis, all infected tissues were excised, and the resultant defects were corrected with patches of decellularized human pericardia.

Except for patients with acute endocarditis, a Carpentier-Edwards Physio II annuloplasty ring^®^ (Edwards Lifesciences LLC, Irvine, CA, USA) or a posterior bovine pericardium band^®^ (Cardioprótese Ltda, Curitiba, PR, Brazil) was employed to correct annular dilatation and to stabilize the repair. Valve competence was tested with saline solution or by pressurizing the left ventricle (LV) with blood through a cardioplegia line inserted in the apex of the LV. Tricuspid annulus dilatation with or without regurgitation (TR) was corrected with Carpentier-Edwards Tricuspid Physio ring^®^ (Edwards Lifesciences, Irvine, CA, USA) and CABG was performed with standard techniques. To ascertain an adequate repair, intraoperative TEE was performed in all patients after weaning from CPB.

### Patient Data and Clinical Follow-up

Preoperative clinical data were obtained by reviewing hospital charts and operative notes. Early mortality was defined as any death occurring before hospital discharge or during the first 30 postoperative days. Causes of early and late deaths were determined by hospital charts review, death certificates, information from the physician who was caring for the patient at that time or communication with the patient's family. Early and late postoperative complications were reported according to well-established guidelines^[6]^.

Clinical follow-up was obtained at one, six, and 12 months after the operation and then annually thereafter in our outpatient clinic, with the referring cardiologist or by direct contact with the patient or his/her family. For this study, a database freeze was performed in March 2017. Clinical follow-up was obtained within two years of closure in 95% of the patients (five patients were lost to follow-up). Anticoagulation with sodium warfarin was indicated only to patients with AF, to those who had AF ablation or after an episode of cerebral embolism. Patients with RD were oriented to have lifelong oral penicillin as a secondary prophylaxis against rheumatic fever.

### Echocardiographic Analysis

Mitral valve function was evaluated by transthoracic twodimensional Doppler echocardiography, performed by the referring cardiologist or at our outpatient clinic at yearly intervals. Whenever it was necessary, supplemental information was obtained with 2D or 3D TEE studies.

All patients were operated with intraoperative TEE control, including 3D real time images in the last two years.

MR was initially classified as none, trivial, mild, moderate, and severe, based on the length and area of the regurgitant jet and using American Society of Echocardiography's guidelines^[7]^. Valve failure was defined as recurrent significant regurgitation of more than 1+ MR (mild MR) or mitral valve reoperation.

### Statistical Analysis

Statistical analysis was conducted with The R Project for Statistical Computing statistical software (www.cran.r-project.org/ version 3.3.1) and Graph Pad Prism Software (www.graphpad.com/guides/prism/7/). Data were presented as frequencies or means with standard deviations. The Kaplan-Meier method was used to estimate survival and freedom from morbid events, and results were considered meaningful up to 10 years. For outcomes other than death, patients were right censored in case of a late death. Age- and sex-matched Brazil general population survival estimates for the year 2013 (median year of surgery of the study cohort) were obtained from data published by the Instituto Brasileiro de Geografia e Estatística (http://www.ibge.gov.br), and statistical comparison with survival rates from the study cohort was done using a one-sample log-rank test.

Univariable analysis was carried out with chi-square tests and log-rank test to determine the risk factors for reoperation and valve failure, and they were considered significant when *P*<0.05. Predictors were expressed by their hazard ratios (HR) with 95% confidence intervals (CI). Variables tested included patient's age, gender, New York Heart Association (NYHA) functional class, etiology, AF, systemic arterial hypertension, chronic obstructive pulmonary disease (COPD), diabetes, smoking, renal failure, coronary artery disease (CAD), previous cardiac operations, concomitant procedures, cross-clamp, and extended extracorporeal circulation (ECC) times.

## RESULTS

From January 2004 to March 2017, our surgical group has performed 538 MVR, of which 125 were done at INC-Cardio and are the subject of this study. During this period, 133 patients were operated with a diagnosis of organic MR, and only eight (acute endocarditis = 3, advanced mitral mixed lesions = 3, and failed previous MVR done elsewhere = 2) had a mitral valve replacement, yielding an overall 93.9% global repair rate. All patients (100%) with degenerative mitral valve disease undergoing a first operation had a MVR.

The demographics of the 125 MVR patients are summarized in Table 1. Mean patient age was 57±15 years (range: 9 - 87 years), 52 (42%) patients were males, and 23 (18%) were older than 70 years. The most common etiology was degenerative disease in 73 (58%) patients, followed by RD in 34 (27%). Preoperatively, 44 (35%) patients were in NYHA functional class III and IV, and the mean left ventricular ejection fraction (LVEF) was 67±8%. MR grade was considered severe in 115 patients (92%) and moderate in the remaining 10 (8%).

**Table 1:** Demography.

Variables	N (%)
Number of patients	125
Age at surgery - mean±SD	57±15
Range, years	9 - 87
Sex, male	52 (41.9%)
Etiology	
Degenerative	73 (58.4%)
Myxomatous degeneration	57 (45.6%)
Fibroelastic deficiency	9 (7.2%)
Barlow syndrome	7 (5.6%)
Rheumatic	34 (27.2%)
Congenital	5 (4%)
Pure annular dilatation	3 (2.4%)
Infective endocarditis	9 (7.2%)
Healed	5 (4%)
Active	4 (3.2%)
Endomyocardial fibrosis	1 (0.8%)
NYHA Class	
I	23 (18.4%)
II	58 (46.4%)
III	36 (28.8%)
IV	8 (6.4%)
Previous cardiac operations	13 (10.4%)
Systemic arterial hypertension	59 (47.2%)
Chronic obstructive pulmonary disease	3 (2.4%)
Diabetes	8 (6.4%)
Smoking	10 (8%)
Renal failure	8 (6.4%)
Coronary artery disease	9 (7.2%)
Atrial fibrillation/Flutter	36 (28.8%)
LVEF (%) - mean±SD	67±8.4
Range	31-84
Below 50%	5 (4%)
DDLV (mm) - mean±SD	55±7.2
Range	41-76
SDLV (mm) - mean±SD	34±6.1
Range	25-56
Mitral regurgitation	
Moderate	10 (8%)
Severe	115 (92%)
Tricuspid regurgitation	
Moderate	18 (14.4%)
Severe	2 (1.6%)

DDLV=diastolic dimension of the left ventricle; LVEF=left ventricular ejection fraction; NYHA=New York Heart Association; SD=standard deviation; SDLV=systolic dimension of the left ventricle

Operative findings, repair techniques, and concomitant procedures are listed in Table 2.

**Table 2:** Operative data and surgical findings.

Variables	N (%)
Number of patients	125
Etiology	
Degenerative	73 (58.4%)
Anterior prolapse	11 (15.0%)
Posterior prolapse	49 (67.1%)
Bileaflet prolapse	13 (17.8%)
Rheumatic	34 (27.2%)
Congenital	5 (4%)
Pure annular dilatation	3 (2.4%)
Endomyocardial fibrosis	1 (0.8%)
Active infective endocarditis	4 (3.2%)
Healed infective endocarditis	5 (4.0%)
Incision	
Median sternotomy	115 (92.0%)
Right minithoracotomy	10 (8.0%)
No annuloplasty ring	11 (8.8%)
Mitral annuloplasty ring	114 (91.2%)
Carpentier-Edwards Physio II Ring	61 (48.8%)
Bovine Pericardial band	47 (37.6%)
Gregori Ring 2	2 (1.6%)
Carpentier Classic Ring	3 (2.4%)
Braile 1	1 (0.8%)
Chordal replacement with Gore-Tex sutures	76 (60.8%)
Use of pericardial patches	12 (9.6%)
Triangular resection	20 (16.0%)
Quadrangular resection	24 (19.2%)
Tricuspid valve surgery	20 (16.0%)
Coronary artery bypass graft	8 (6.4%)
Atrial fibrillation ablation	18 (14.4%)
CPB time (min) - mean±SD	93±35
Range	35 - 240
Aortic clamping time (min) - mean±SD	73±29
Range	23 - 197

CPB=cardiopulmonary bypass; SD =standard deviation

The mean clinical follow-up was 3.7±3.4 years (range: 0.1 - 12.4 years), with a total cumulative follow-up of 462.2 patientyears. In total, 697 echocardiograms were available for analysis. The latest echocardiogram was performed after a mean of 3.1±2.9 years (range: 0.1 - 12.3 years).

### Early and Late Mortality

There were three early deaths with an overall early mortality of 2.4%. Among patients younger than 70 years, early mortality was 0.9% (1/102). Causes of early death were multi-organ failure in an 81-year-old patient with degenerative disease and healed bacterial endocarditis, respiratory failure in a 77-year-old patient with degenerative disease and low output syndrome in a rheumatic patient that was submitted to concomitant myocardial revascularization and AF ablation.

**Fig. 1:**
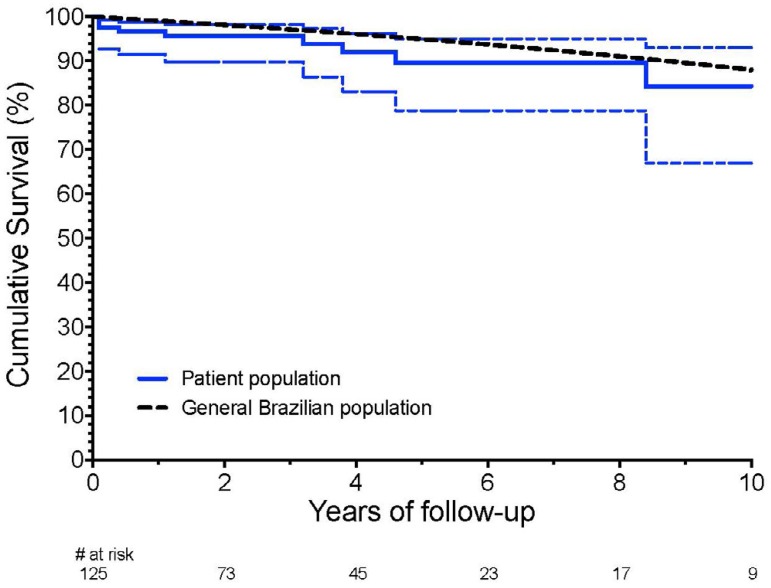
Late survival after mitral valve repair.

In addition, six patients died in the late postoperative period. Causes of late death were sudden death (n = 2), congestive heart failure, intracranial hemorrhage, hepatic cirrhosis, and unknown, in one case each. By Kaplan-Meier analysis, estimated five and 10 years survival were 89.5% (CI 95% = 78.8% - 94.9%) and 84.3% (CI95% = 67.1% - 92.9%), respectively, which were similar to an age- and gender-matched Brazilian population (Figure 1).

Risk factors for late mortality included age (HR = 3.30, CI95% = 0.85 - 12.7), associated CAD (HR = 2.48, CI95% = 0.42 - 14.2), diabetes (HR = 7.85, CI95% = 0.49 - 123.4), longer aortic crossclamp (HR = 4.07, CI95% = 1.09 - 15.1), and ECC times (HR=5.00, CI95% = 1.17-21.2) (Appendix 1).

### Clinical Follow-up

**Fig. 2:**
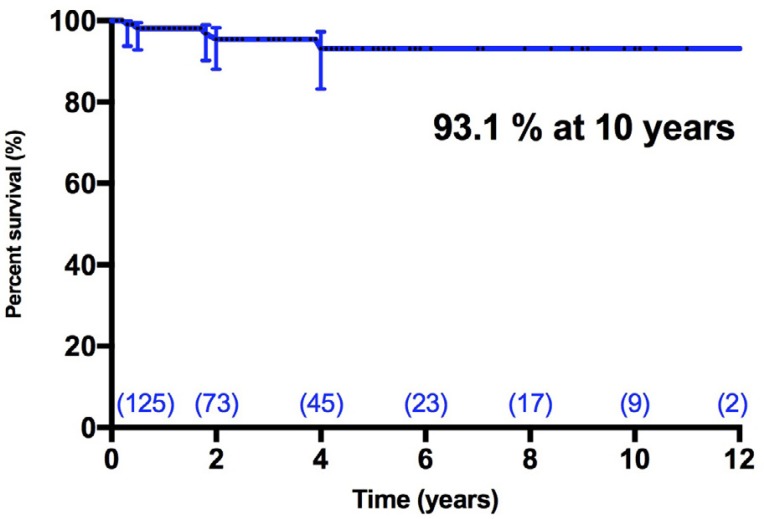
Freedom from thromboembolic complications after mitral valve repair.

Among the 111 survivors with known clinical status, 101 are in NYHA functional class I, nine in class II and only one in class III. This latter patient, despite a normally functioning mitral repair, developed moderate to severe tricuspid regurgitation and should undergo reoperation in the near future. During the observation period, five patients presented thromboembolic events, three were transient ischemic attacks and two were strokes. Freedom from thromboembolic events at five and 10 years was 93.1% (CI95% = 83.3% - 97.2%) (Figure 2). In addition, two patients presented with serious hemorrhagic complications, which were the cause of death in one. There were no documented cases of bacterial endocarditis.

By echocardiogram, late LVEF was 65.5±7.5% (range = 27 - 77), with only three being below 50%. Late diastolic dimension of the left ventricle (DDLV) was 48±6 mm (range = 37 - 74) and systolic dimension of the left ventricle (SDLV) was 31±6 mm (range = 22 - 68).

### Reoperations and Mitral Valve Dysfunction

At discharge, only one patient had moderate MR with no further progression after six years.

**Fig. 3:**
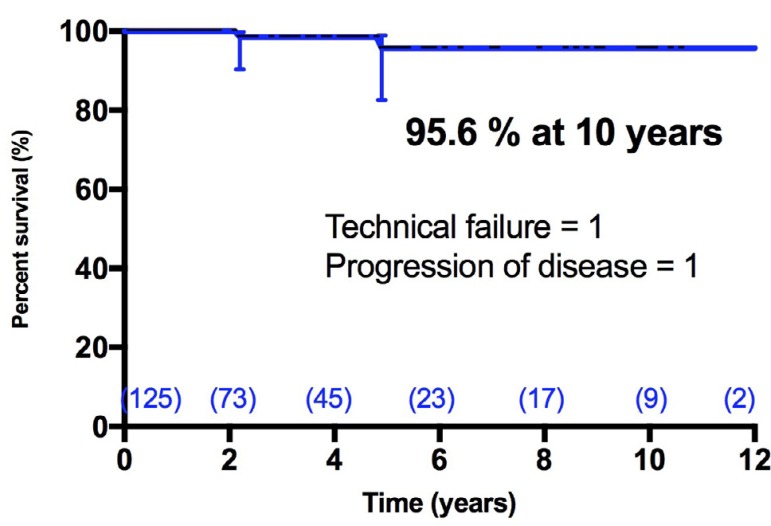
Freedom from severe mitral regurgitation (MR) after mitral valve repair. Two patients developed this complication, and both were reoperated.

During follow-up, two patients developed severe MR and were reoperated. Mitral valve could be re-repaired in both. Mechanisms of failure were dehiscence of the posterior leaflet suture line in one case (technical failure) and progression of the disease in another, with a new prolapse of the anterior leaflet. Freedom from severe MR and/or reoperation was 95.6% (CI95% = 82.5% - 98.9%) at 5 and 10 years (Figure 3).

**Fig. 4:**
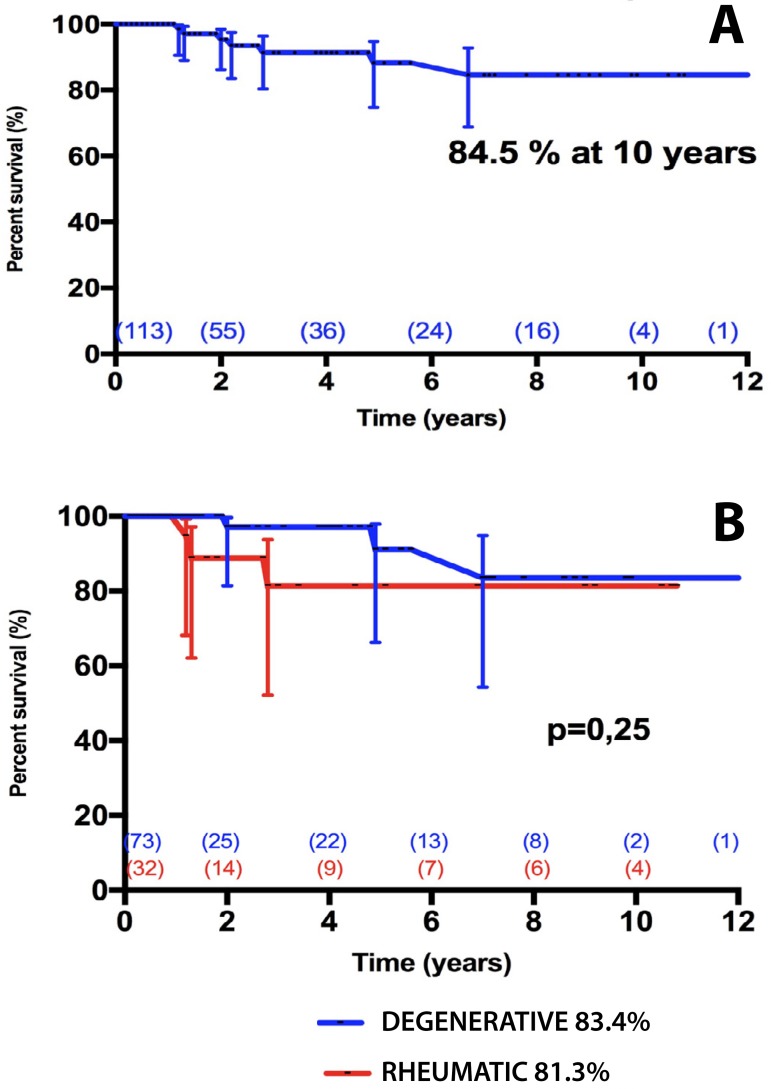
A) Freedom from valve failure after mitral valve repair. Failure was defined as any mitral regurgitation (MR) ≥ moderate or reoperation due to any cause. B) Freedom from valve failure stratified according to patients presenting with degenerative or rheumatic disease. MR=mitral regurgitation

Four additional patients developed moderate MR at late follow-up, but they are asymptomatic and under careful observation. Freedom from valve failure (more than mild MR or reoperation) was 88.2% (CI95% = 74.8% - 94.7%) at 5 years and 84.5% (CI95% = 68.8% - 92.6%) at 10 years. There was no difference in freedom from valve failure between degenerative and rheumatic valves at 10 years (Figure 4). Curiously, univariable analysis revealed renal failure as the only risk factor for late mitral valve dysfunction (HR = 9.31, CI95% = 0.22-397.66) (Appendix 1).

### Tricuspid Valve Function

**Fig. 5:**
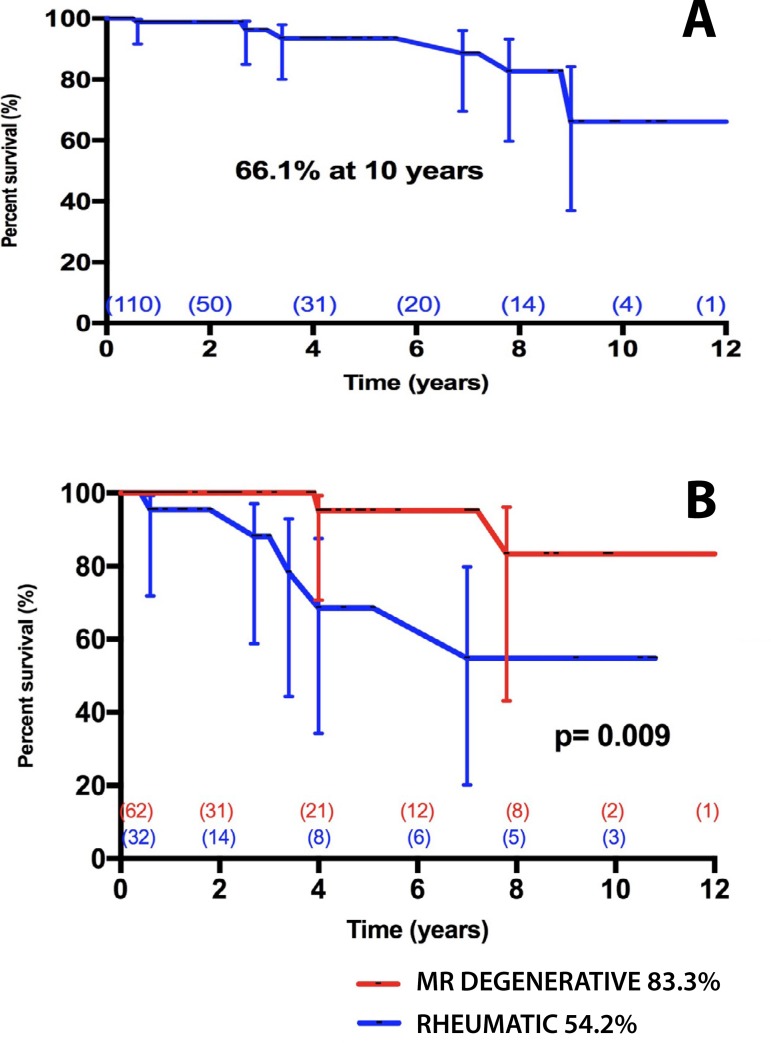
A) Freedom from late development of moderate or severe tricuspid regurgitation (TR) after mitral valve repair. B) Freedom from late development of moderate or severe tricuspid regurgitation (TR) after mitral valve repair stratified according to patients presenting degenerative or rheumatic disease. MR=mitral regurgitation

Four patients (moderate = 3, severe = 1) with preoperative TR underwent concomitant tricuspid annuloplasty during the operation. In addition, 16 patients with dilated tricuspid annulus, but with none or mild TR, also underwent "prophylactic"tricuspid repair with a Carpentier Edwards Tricuspid Physio ring^®^. None had more than mild TR at late follow-up. In contrast, new moderate (n = 6) or severe TR (n = 1) was detected in patients in whom the tricuspid valve was not addressed during the primary operation. Overall freedom from ≥ moderate TR was 66.1% (CI95% = 37.1 - 84.2%) at 10 years. Univariable analysis revealed RD as the only risk factor for development of late TR (HR = 6.69 [CI95% = 1.69 - 40.09] - P=0.044) (Figure 5, Appendix 1).

## DISCUSSION

This study demonstrates that it is possible to obtain high rates of MVR for patients with organic MR, from different etiologies, in dedicated centers^[8,9]^. Our repair rate of 93.9% for all-comers includes not only patients with degenerative disease, but also those with more challenging rheumatic and acutely infected valves^[10,11]^. This aspect seems to be very relevant, at a time when the American College of Cardiology (ACC) and other cardiological societies are making efforts to create high volume regional reference centers that fulfill excellence criteria in the surgical management of patients with MR. It is our opinion that in Brazil, where global reparability rates are still very low, special programs such as specialized fellowships and dedicated symposiums should be promoted for adequate training of surgeons, clinicians, anesthesiologists and echocardiographists.

Several groups have documented excellent outcomes and a reparability rate ranging from 95-100% for patients with degenerative disease^[12-16]^. In the present series, 97.2% of degenerative valves were treated in this manner, with very acceptable early mortality and low incidence of residual regurgitation. The only two patients who had mitral valve replacement were cases with previous repairs done elsewhere and which were judged as suboptimal candidates for a second repair due to important scaring and distortion of the valve apparatus.

On the other hand, rheumatic and/or infected valves may impose special challenges, and reports about conservative surgical treatment in these situations have demonstrated a much lower reparability rate^[10,11]^. We believe, however, that with proper surgical expertise and an aggressive approach, it is possible to avoid valve replacement in a substantial number of cases in this subset of patients.

Our overall early mortality of 2.4% was acceptable in view of the wide range of pathologies treated and not limited to the more simple degenerative posterior mitral valve prolapse^[8]^. Our three deaths occurred only in older patients with extensive degenerative disease or in the presence of associated CAD. Mortality was 0% for patients with isolated primary mitral valve surgery, with or without concomitant tricuspid or AF, under the age of 70 years, which compares favorably with the STS database^[5]^.

A relevant finding of this study was the excellent long-term survival, that was similar to the age- and sex-matched Brazilian population at least for the first decade after operation. This reinforces our tendency for recommending early surgery for patients with severe MR, even for asymptomatic patients, before they reach class I guideline triggers for surgery, such as AF and pulmonary artery hypertension^[17]^. Although some authors feel that a "watchful waiting" policy is safe and reasonable for asymptomatic patients^[18]^, recent studies have demonstrated that long-term survival may be compromised with that approach^[19,20]^.

Not only survival, but also long-term functional results were very gratifying, with most patients presenting normal functional recovery in NYHA class I and with low incidence of valve related complications. Thromboembolic events were uncommon, occurred more frequently during the first few years and had no relation with the presence of AF. However, it must be emphasized that all patients with documented AF were under anticoagulation therapy. Furthermore, 16 out of 18 patients who had AF treated by bipolar radiofrequency are in sinus rhythm as demonstrated by regular electrocardiograms and/or 24 hours Holter examination. It is also important that there were no cases of bacterial endocarditis during the observation period, even in patients that had acute or healed endocarditis as the primary reason for operation. Although this study did not intend to compare MVR with mitral valve replacement, one should expect a higher incidence of valve-related complications if these patients have had replacement as the surgical procedure^[1,2,21]^.

One of the caveats of reconstructive mitral surgery is the possibility of recurrent MR and eventual need for reoperations. The main reasons for recurrent MR are technical failures, disease progression and scarring after the repair^[22]^. In this study, because the incidence of recurrent severe MR was infrequent and the number of patients was relatively small, statistical analysis had important limitations for establishing risk factors associated with this complication. One important finding, however, was that, at least up to ten years, results in rheumatic patients were similar to those with degenerative disease. We have been very aggressive in treating rheumatic pathologies, making extensive shaving of thickened and retracted tissues, debridement of calcified areas, cutting secondary chords and even performing partial and total primary chordal replacement to increase leaflet mobility and to obtain adequate coaptation surface areas. In the presence of retracted anterior leaflets, cusp extension with decellularized pericardium has been an important maneuver for a satisfactory result^[23]^. With that policy, the incidence of recurrent moderate or severe MR has been low, although we often must accept smaller final effective orifice areas (around 1.8 - 2.5 cm^2^) in patients with mixed lesions. In our country, Severino et al.^[24]^ and Pomerantzeff et al.^[25]^ have also shown the apparent feasibility and advantages of MVR in rheumatic patients. On the other hand, it must be emphasized that most rheumatic patients in this study had the so-called "burn-out" disease, that is less susceptible to newer acute inflammatory bursts of rheumatic fever, besides being carefully oriented and controlled with lifelong antibiotic prophylaxis against the disease^[11]^.

Although still controversial, several recent reports have stressed the importance of avoiding late tricuspid regurgitation after a successful mitral operation^[26-29]^. It is becoming more apparent that the concept that any degree of functional tricuspid regurgitation would improve by correcting left-sided lesions only is misleading, and the occurrence of moderate to severe TR and eventual need for reintervention is not negligible^[26]^. This is corroborated in the present series in which approximately one third of the patients had more than mild TR late after the initial operation when the tricuspid valve was not addressed, especially in rheumatic patients. Furthemore, reoperations for isolated late TR after MVR carry a high operative risk and thus should be avoided^[27]^. In the initial phase of the present series, we have repaired the tricuspid valve whenever we found moderate or severe TR with symptoms of right side failure and visual right ventricular (RV) and right atrial (RA) enlargement during the operation. More recently, however, we moved towards a more aggressive approach on the tricuspid valve, and we performed tricuspid annuloplasty not only in patients with moderate or severe functional TR but also when dilatation of the tricuspid annulus was greater than 40 mm by echocardiography^[30]^, as recommended by Chikwe et al.^[26]^ and Dreyfus et al.^[28]^. Although follow-up is still short, we have not identified a single late TR after tricuspid annuloplasty with Carpentier-Edwards Tricuspid Physio ring.

This study has several limitations. All operations were performed by a single surgeon and selection biases and individual approaches to certain pathologies may influence outcomes, and the results may not be generalized. In the first years of this experience, tricuspid valve annulus size and degree of regurgitation were not evaluated in the same systematic manner as more recently, so underestimation of TR may have occurred. Furthermore, because TR is not a terminal event and longitudinal echocardiography data are not complete, any conclusion regarding the true incidence of more than mild TR should be done with caution. Some echocardiography data regarding late function of the mitral and tricuspid valves were obtained outside our clinic and may cause some inconsistencies.

## CONCLUSION

In conclusion, we have demonstrated that MR due to organic mitral valve disease, from various etiologies, can be surgically treated with a high repair rate, low early mortality and long-term survival that are comparable to the matched general population. Concomitant treatment of AF and tricuspid valve may be important adjuncts to optimize long-term results. In our opinion, however, this can only be accomplished in reference centers with a dedicated heart valve team working in a focused and systematic way in order to obtain consistent results.

**Table:** 

Authors' roles & responsibilities	
FDAC	Substantial contributions to the conception or design of the work; or the acquisition, analysis, or interpretation of data for the work; final approval of the version to be published
DFFC	Substantial contributions to the conception or design of the work; or the acquisition, analysis, or interpretation of data for the work; final approval of the version to be published
GLSM	Substantial contributions to the conception or design of the work; or the acquisition, analysis, or interpretation of data for the work; final approval of the version to be published
KCSP	Substantial contributions to the conception or design of the work; or the acquisition, analysis, or interpretation of data for the work; final approval of the version to be published
MCB	Substantial contributions to the conception or design of the work; or the acquisition, analysis, or interpretation of data for the work; final approval of the version to be published
EMBF	Substantial contributions to the conception or design of the work; or the acquisition, analysis, or interpretation of data for the work; final approval of the version to be published
MV	Substantial contributions to the conception or design of the work; or the acquisition, analysis, or interpretation of data for the work; final approval of the version to be published
GM	Substantial contributions to the conception or design of the work; or the acquisition, analysis, or interpretation of data for the work; final approval of the version to be published
ADAF	Substantial contributions to the conception or design of the work; or the acquisition, analysis, or interpretation of data for the work; final approval of the version to be published
CC	Substantial contributions to the conception or design of the work; or the acquisition, analysis, or interpretation of data for the work; final approval of the version to be published

## Appendix 1.

**Table:**

Risk factors for mitral valve dysfunction	Hazard ratio (95%CI)	*P*-value
Age	0.455 (0.085 to 2.423)	0.453
Sex	0.259 (0.056 to 1.182)	0.177
NYHA functional class	1.856 (0.413 to 8.337)	0.409
Atrial fibrillation	1.024 (0.197 to 5.329)	0.976
Systemic arterial hypertension	1.571 (0.355 to 6.950)	0.549
Chronic obstructive pulmonary disease	0	0.624
Diabetes	3.121 (0.106 to 91.445)	0.265
Smoking	2.121 (0.122 to 36.685)	0.475
Renal failure	9.315 (0.223 to 387.661)	0.001
Coronary artery disease	0	0.645
Previous cardiac operations	3.357 (0.303 to 37.079)	0.123
Associated surgical procedures	1.939 (0.371 to 10.118)	0.375
Etiology	2.033 (0.382 to 10.798)	0.342
Cardiopulmonary bypass time	0.959 (0.188 to 4.873)	0.960
Aortic clamping time	0.732 (0.155 to 3.451)	0.705

CI=confidence interval; NYHA=New York Heart Association

**Table:**

Risk factors for mortality	Hazard ratio (95%CI)	*P*-value
Age	3.301 (0.857 to 12.709)	0.042
Sex	1.979 (0.535 to 7.312)	0.290
NYHA class	1.552 (0.390 to 6.163)	0.491
Atrial fibrillation	0.871 (0.224 to 3.384)	0.824
Systemic arterial hypertension	1.753 (0.473 to 6.495)	0.391
Chronic obstructive pulmonary disease	0	0.682
Diabetes	7.855 (0.499 to 123.422)	0.000
Smoking	0.563 (0.103 to 3.065)	0.404
Renal failure	2.546 (0.115 to 56.145)	0.356
Coronary artery disease	2.485 (0.432 to 14.295)	0.029
Previous cardiac operations	3.647 (0.294 to 45.196)	0.079
Associated surgical procedures	1.863 (0.491 to 7.065)	0.292
Etiology	0.430 (0.090 to 2.055)	0.400
Cardiopulmonary bypass time	5.002 (1.177 to 21.245)	0.007
Aortic clamping time	4.076 (1.099 to 15.115)	0.034

CI=confidence interval; NYHA=New York Heart Association

**Table:**

Risk factors for tricuspid valve dysfunction	Hazard ratio (95%CI)	*P*-value
Age	1.120 (0.115 to 10.844)	0.918
Sex	0	0
NYHA class	2.422 (0.398 to 14.705)	0.307
Atrial fibrillation	1.749 (0.246 to 12.431)	0.530
Systemic arterial hypertension	4.339 (0.750 to 25.085)	0.150
Chronic obstructive pulmonary disease	NaN	1
Diabetes	0	0.622
Smoking	0	0.734
Renal failure	0	0.773
Coronary artery disease	0	0.872
Previous cardiac operations	0	0.455
Associated surgical procedures	0.730 (0.097 to 5.454)	0.776
Etiology	6.698 (1.094 to 40.998)	0.044
Cardiopulmonary bypass time	0	0.321
Aortic clamping time	0	0.287

CI=confidence interval; NYHA=New York Heart Association
